# Supplementary material for: Purkinje cells of the cerebellum control deceleration of tongue movements
Source: PLoS Biol. 2025 Apr 10;23(4):e3003110. doi: 10.1371/journal.pbio.3003110 (PMC11984719; doi:10.1371/journal.pbio.3003110)
Supplement: S1 Table — (DOCX) [file pbio.3003110.s018.docx]

**S1 Table. Distribution of tongue and eye modulated P-cells in the vermis region of the cerebellum**

| **Region** | **Eye Modulated** | **Tongue Modulated** | **Only Eye Modulated** | **Only Tongue Modulated** | **Both** | **Neither** | **Saccade z-score** (mean ± SD) | **Lick z-score** (mean ± SD) |
| --- | --- | --- | --- | --- | --- | --- | --- | --- |
| **Lobule VI** | 49/98 (50.00%) | 97/98 (98.98%) | 0/98 (0.00%) | 48/98 (48.98%) | 49/98 (50.00%) | 1/98 (1.02%) | 3.52 ± 2.05 | 7.67 ± 3.52 |
| **Lobule VII** | 56/59 (94.92%) | 57/59 (96.61%) | 2/59 (3.39%) | 3/59 (5.08%) | 54/59 (91.53%) | 0/59 (0.00%) | 8.21 ± 5.39 | 10.06 ± 4.64 |
