# Supplementary material for: Purkinje cells of the cerebellum control deceleration of tongue movements
Source: PLoS Biol. 2025 Apr 10;23(4):e3003110. doi: 10.1371/journal.pbio.3003110 (PMC11984719; doi:10.1371/journal.pbio.3003110)
Supplement: Attachment — Submitted filename: PLOS Biology reviews 1.docx [file pbio.3003110.s021.docx]

**Major additions to the paper:**

1) Addition of figures that quantify effects of long pauses in the simple spikes. These new results confirm that SS pauses, even without a preceding complex spike, are followed by forces that pull the tongue outward.
2) Addition of new figures that show complex spike patterns during successful and unsuccessful licks. These new results demonstrate that lick errors induce complex spikes.
3) Addition of figures that show modulation of P-cells during both eye and tongue movements and compare modulation strength in each cell during these two behaviors.
4) Addition of 9 videos that display the various types of licks, including licks that miss the target, along with kinematic measures for each video that track the various locations on the tongue and display speed and distance.
5) Addition of figures that show modulation of simple spikes as a function of tongue endpoint throughout the workspace.

**Reviewer #1** (Detlef H. Heck):

The manuscript by Hage et al. describes interesting findings from well-designed experiments to determine the influence of complex spike-induced changes in Purkinje cell simple spike activity on goal directed tongue movements in marmosets. The strength of the approach chosen is that recordings are performed in a primate and the authors did not manipulate large populations of Purkinje cells with population synchronizing optical, chemical or electrical stimulations but instead recorded naturally occurring instances of complex spikes during a large number of licks performed to gain food rewards. The results are in surprising in the sense that a complex spike observed in just a single Purkinje cell (causing the expected brief pause in simple spike activity) had a significant effect on tongue protrusion and retraction if it occurred during certain phases of the tongue movements, and that this effect was significantly increased when a complex spike
 occurred simultaneously in two observed Purkinje cells. The overall direction of the effect on tongue movements indicates that simple spike activity serves to slow down tongue movements to properly stop at the desired target, which is a finding that resonates with cerebellar involvement in both eye or limb movements.

There are no major concerns with this manuscript. The experimental design and data analysis are solid, and the manuscript is well written with some minor issues.

Minor concerns:

**The figures are not cited in order throughout the text. The authors should rearrange the figures to fix this issue.**
Fixed.

**The recording location is mentioned early in the manuscript but should also be mentioned in the method section, where it just says "cerebellum".**
Fixed

**Complex overlapping bar graphs (e.g. Fig. 2 C) are sometimes difficult to read. Consider making them larger.**
Done

**The tongue trajectory plots in Fig. 3 A and G are difficult to interpret. Maybe enlarge them or describe better what they are showing.**
We added significant new text to the figure legend to clarify the plots, and refined the figure to highlight how the tongue trajectory was changed following the P-cell suppression.

**Legend for Fig. 3A refers to an "orange bar" that this reviewer cannot find in the figure.**
Fixed.

—-----------------------------

**Reviewer #2:**

In this manuscript, Hage et al. present their research on cerebellar control of tongue movements in marmosets. The study focuses on how vermal Purkinje cells (P-cells) contribute to the precision of tongue movements during food harvesting. Using electrophysiological recordings and high-speed video tracking, the authors analyzed the relationship between complex spikes (CS), simple spikes (SS), and tongue kinematics. They found that CS-induced pauses in SS firing led to hypermetria during protraction and slowing during retraction of the tongue. The hypermetria increased with synchronous CS firing across multiple P-cells, demonstrating the importance of synchronous CS firing. Surprisingly, this phenomenon was consistent across the recorded P-cells despite heterogeneity in their SS firing patterns. Finally, they explored the encoding of kinematic parameters by SS rate, which showed significant modulation by peak velocity and duration of deceleration. Notably, this modulation became much less significant when the tongue movement was not directed toward an external goal (food) but was used for grooming.

This is an interesting study with many fascinating findings based on a novel paradigm. However, I found several confusing points that need clarification. Also, the authors need to improve their presentation of the data, which can help with clarification.

**Major issues:**

**1. P-cell selectivity to tongue movement. The data is obtained from the vermis including lobule VI/VII during animals performing the saccade task. An important question here is whether the P-cells in this dataset were eye movement-sensitive, and, if so, how many of them are in the dataset. Regardless of whether the answer is yes or no, this would be an interesting finding to report.**

Thank you for the suggestion. We added the new results in Supplementary Fig. S2 (below). Our subjects typically performed reward relevant saccades for 5-10 consecutive trials, then stopped performing the saccade task, fixated the tube, and harvested the food that had accumulated. This allowed us to distinguish the simple spike modulations during reward relevant saccades, and reward relevant licks. The results for two representative P-cells are plotted in Supplementary Fig. S2C, and summarized in Supplementary Fig. S2B. In our dataset, the SS modulations were usually present for both tongue movements and eye movements. However, as a population the P-cells were more strongly modulated by tongue movements (paired t-test, t(156)=7.96, p=3.3E-13). This differential encoding of tongue vs. eye was stronger for neurons that were located in lobule VI (Supplementary Fig. S2B).

In addition, we added the following table to the supplementary data.

**2. SS pauses. This study focuses on pauses in SS firing caused by CSs. CSs induced short pauses of ~15 ms while they are associated with significant differences in tongue movements. However, P-cells can pause SS firing without CSs, contributing to multiplexing different types of information into the P-cell SS output similar to the CS-induced pauses in this study. Therefore, it is necessary to ve(Hong et al., eLife, 2016; Brown et al., Neuron, 2024). Furthermore, there is evidence that those SS pauses synchronously begin or terminate across multiple P-cells (Jaeger, J Comp Neurosci, 2003; Shin and De Schutter, J Neurophys, 2006; Hong et al., eLife, 2016), rify whether the changes in the tongue movements are specific to the CS-driven pauses or general SS pauses. Specifically, I suggest analyzing how the distribution of pause lengths after CSs (~15ms) compares to that of all SS interspike intervals (ISIs) to verify if there are large ISIs that interrupt fast simple spiking, or SS pauses. If so, the authors should test if non CS-driven pauses can evoke changes in the tongue movements. Regardless of what the authors find from this analysis, it would be valuable information.**

Thank you for the suggestion. We performed this analysis and included a new section in the paper that describes the results. We found that regardless of whether an SS pause was due to the arrival of a CS or not, the downstream effects were the same: production of forces that extended the tongue, bending it away from the midline. The added section, and the new figure, are noted below.

*Simple spike pause without a complex spike was sufficient to produce hypermetria*

We interpreted the kinematic effects that followed the CS-induced SS suppression as being due to SS suppression, not due to the presence of a CS. To check the validity of this conjecture, we quantified the kinematic effects of non-CS-induced long SS pauses on the tongue’s trajectory. To identify a long SS pause, for each P-cell we considered all licks towards the same direction in which no CS occurred at any point in the movement. We then identified the longest inter-spike interval (ISI) for the SSs in each phase of each lick (phase refers to protraction deceleration period, etc.). For each phase under study, and each P-cell, we labeled 25% of the licks with the largest ISIs as a “long-pause” lick.

Next, we considered triplets of consecutive licks {n-1, n, n+1} of the same type in the same direction in which none of the licks had a CS during any phase of the movement. We selected the subset of triplets in which lick n had a long pause in only one phase of the movement, but no long pauses in any phase of the neighboring licks. We then compared tongue trajectories between the lick that had a long SS pause with its two neighboring licks.

On average, the duration of a long SS pause was xxx+/-yy ms (mean+/-SEM). If this event occurred during the protraction acceleration period, it produced hypermetria and bending of the tongue away from the midline (Fig. 6A), and if it occurred during the retraction period it produced a slowing of the return and also a bending away from the midline (Fig. 6B). The trajectory changes were quite similar to what we had observed following a CS-induced SS suppression.

Thus, regardless of whether an SS pause was due to the arrival of a CS or not, the downstream effects were the same: production of forces that extended the tongue, bending it away from the midline.

The effect of SS pause without a preceding CS.

**3. Data presentation. I had some difficulty understanding the left and right panels in Fig. 1D showing different segments in the recording data.**

We added labels to the figures, identifying the bout onset figure and labeling the licks during the bout.

**Additionally, Fig 5A and other figures seem to suggest that P-cell activity is quasi-periodic as the animal repeats similar tongue movements. Was there any reason not to use a cyclic description (e.g., amplitude & phase) of tongue movement for analysis? It would be beneficial if the authors could comment on whether the tongue movements were sufficiently cyclic or not.**

Indeed the behavior was quasi-periodic. We added a new supplementary figure to show lick kinematics in much greater detail (see below). However, our aim was to ask whether SS suppression affected the movement differently during the various phases of the lick. To answer this, our analysis focused on dividing the protraction phase into the acceleration and deceleration periods, which critically depended on the timing of the peak velocity on each lick. Because the first licks was typically slower than the second licks, then sped up during the bout, and finally slowed down as the bout ended (Fig. 5A), we chose not to average across the licks using a phase analysis, but rather align the licks to specific time points: lick onset, protraction peak velocity, and retraction onset.

**4. Encoding of kinematic parameters by SSs. The authors' explanation about the longer deceleration case from lines 304 to 308 is quite confusing. If the motion starts with a certain initial speed but takes longer to decelerate until stopping, one would expect the magnitude of deceleration and therefore the force (and consequently the SS rate according to the authors' interpretation) to be smaller. Additionally, the travel distance would be longer in this case, which seems consistent with the increased distance to the mouth observed with the SS pause (rate decrease). Maybe I am misreading this passage, but it certainly needs clarification to avoid misinterpretation.**

We wanted to test the idea that the simple spike rates during protraction were related to the forces that are needed to decelerate the tongue. We tested this idea in two ways. First, we considered licks that had the same amplitude but different peak velocities (high vs. low vigor). Second we considered licks that had the same peak velocity but different amplitudes (high vs. low amplitude, corresponding to short vs. long deceleration period). We clarified this idea in the presentation of the results and in the figure legend.

To explore how P-cell SS rates encoded the kinematic parameters of lingual movements, we visualized PC firing rates as a function of tongue endpoint position. The firing rate of each P-cell at maximal tongue velocity was computed on a trial-by-trial basis and associated with the spatial coordinates corresponding to the endpoint of that lick. Single trial spike data was smoothed with a Savitzky-Golay filter. Spatial coordinates were standardized across animals such that all contralateral licks appear to the left and all ipsilateral licks to the right. A 100x50 grid was mapped onto the full range of tongue endpoint values, and the summary firing rates at each point were estimated using a natural neighbor interpolation, effectively weighing contributions of neighboring firing rate values based on proximity. The interpolated surface was then smoothed with a 2-D Gaussian filter to produce a continuous heatmap. To ensure population-level robustness of firing rate values, a cell coverage mask was then applied over the heatmap, removing any grid points that did not have at least 75% of the available PCs (118/157 SS cells) contributing to the data. This subfigure was added to Fig. 7 (below).

Minor issues:

 **1. In Fig. 1B and D, small numbers (possibly data IDs or version numbers) should be removed.**

We opted to keep this as it provides information regarding which cell we used to plot the data.

**2. The authors state neurons were recorded in "various locations" in the vermis including lobule VI and VII. Can they clarify this by describing how the recording locations are distributed? How many neurons were confirmed to be in lobule VI/VII? A more precise breakdown of recording locations would be helpful.**

We show neuron counts per lobule as well as a corresponding measure of lick vs saccade related activity. Lobule targeting was achieved by utilizing subject specific imaging and electrode alignment techniques previously described in Sedaghat-Nejad et al., 2019.

—-----------------------------

**Reviewer #3** (Jesse Goldberg):

This paper examines Purkinje cell discharge patterns in a dexterous lick task designed for marmosets.

Major strengths:

1) Paper is very well written.
2) The behavioral task and choice of model system to examine tongue control is innovative and well executed/justified. Natural tongue dexterity is leveraged in this well designed task.
3) The authors did rigorous mapping to identify the part of the vermis where they seemed to be 'swimming' in lick related signals. This is important and difficult work that significantly advances the field and sets the stage for future work.
4) The authors identified consistent patterns of Prk and CS activity that were associated with important aspects of lick kinematics. P cell suppression was associated with advancement of the tongue during both protrusion and retraction phases; SS increases were like associated with slowing the tongue during protrusion, likely aiding in endpoint accuracy. This appears to mesh well with past work in primate eye and mouse limb.

Recommendations for improvement

**1) A fuller characterization of lick kinematics (lick angle, speed, etc.) and performance (tube miss rate) for different lick types (types 1 - 5 in Fig 1a) would be helpful.**

We added a new supplementary figure to fully describe the kinematics of the various types of licks in each subject. Velocity, amplitude, and angle were largest for inner tube licks. Protraction was generally much longer in duration than retraction.

**2) With respect to lick types 2 and 4, how do the authors separate licks precisely aimed to the edge of the tube versus a missed lick (e.g., if lick types 1 and 5 missed the spout)?**

We added the text below to the Methods.

Licks were categorized based on heuristics that considered the position of the tongue relative to the tube opening and the food. We tracked 4 regions of the tongue consisting of the tip, the midpoint, and the left and right edges. The midpoint was computed based on the distance between the tip marker and the opening of the mouth, while the left and right edges were computed based on the mid-distance between the tip and midpoint, positioned at max laterality. Furthermore, we tracked the left and right edges of the opening of each reward tube as well as the densest edge of the food contained within.

Licks were labeled as reward seeking when the region of the tongue within the marker coordinates overlapped with the region of the tube within the edge marker coordinates. Alternatively, licks were labeled as grooming when no overlap occurred.

Reward seeking licks were further labeled into subcategories, consisting of inner-tube and outer-edge licks. Inner-tube labels were assigned when the tip, left, and right tongue markers remained within the bounds of the tube markers. Outer-tube labels were assigned when at least one marker exited the tube boundaries, conditioned on the tip having remained within at least 5 mm of the tube opening.

Additional labels were assigned to each reward seeking licks, categorizing them as either successful or failed licks. In all cases, overlap with food dictated these labels. Thus, to call a given lick a ‘missed’ lick, the position of the food within the tube, relative to the tongue, is considered. For example, consider a scenario where the reward in a given tube is depleted, and it is required that an animal initiate an inner-tube lick to acquire a remaining bolus. If the animal initiates a lick with an end-point criteria that assigned it an under-tube-fail label, then that is considered a missed lick. Inversely, however, in a scenario where the tube is at full capacity and the same lick is assigned an under-tube-successful label, then it is not a missed lick. Thus, the position of the tongue markers relative to both the tube edges and reward is considered when inferring outcome of a lick.

Protraction was defined as lick onset to max displacement. Max displacement was defined as the maximum distance of the tip marker from the mouth (coincides with speed minima, i.e., end point). Retraction onset is from max displacement to lick offset.

**3) An image or video of 'task irrelevant' licks, along with each lick type, would be helpful.**

We added 10 videos that contain the raw images as well as the associated kinematic variables that show change as a function of time during one or multiple licks.

**4) How were protraction and retraction defined? By speed minima, maximum lateral displacement, or some other metric? Perhaps this information is buried in the methods but I could not find it - this is an important issue to clarify key aspects of licking and I recommend clarification of this issue reside in the main text.**

Thank you for the question. We added the following to the Methods section: Protraction was defined as lick onset to max displacement. Max displacement was defined as the maximum distance of the tip marker from the mouth (coincides with speed minima, i.e., end point). Retraction onset is from max displacement to lick offset.

**5) What sensory modalities can marmosets use to find the food tube? Can the animals see the food tube, or purely by touch/miss information? Were the eyes tracked during food acquisition as well as during the saccade task?**

We measured eye movements during the entire recording session, including the lick bouts. Indeed, the monkeys could readily see the tubes and tended to fixate the tubes during licking. We now note this fact in the Methods section.

**6) Are the authors missing a panel in Fig. 2 or is there an extra figure legend? There are only 4 figure panels (A -D), but 5 figure legends (A - E).**

Fixed.

**7) Given that the tube miss rate was not described, I would guess that on some occasions, animals would miss the tube in a task-relevant lick, perhaps during CS suppression conditions described in Fig 3. Did the CS responses differ depending on hits vs. misses of the tube around the time of touch?**

We added a new results section that considers errors in lick trajectories, finding that the error event was transmitted to the cerebellum via the climbing fiber input, resulting in complex spikes. Here are the added text and figures.

Inserting the tongue into the tube required precision because the opening was only slightly larger than the tongue. As a result, roughly 15% of the licks failed to enter the tube and did not touch the food (Supplementary Fig. S5). These were licks in which the food was inside the tube, but the tongue failed to enter it (please see the 3 added videos that show examples of these errors). In these unsuccessful licks the tongue did not bend enough and instead hit the tube’s outer edge. Did these failures result in complex spikes?

To visualize the complex spike patterns as a function of the spatial location of the tongue, we needed to compute Pr(CS|x), i.e., the probability of producing a complex spike given that the tongue was at a given location. To arrive at this variable, we used Bayes rule. We began with computing p(x(t-25)|CS(t)), i.e., given that a CS occurred at time t, the likelihood of the tongue’s tip location at time t-25ms. We found that this likelihood depended on whether the tongue successfully entered the tube or not. For licks that were successful and entered the tube, the likelihood was bimodal, exhibiting a peak near lick onset, then a second peak near the food (Fig. 8A, left column). For licks that were unsuccessful and did not enter the tube, the likelihood was also bimodal, but now the second peak was around the edge where the tongue had collided with the tube (Fig. 8B, left column).

However, from these likelihoods we could not infer the probability of complex spikes because p(x) was not uniform (tongue velocity was not constant throughout the lick). Thus, we computed the marginal value p(x) (Fig. 8A and 8B, middle column) and the prior probability Pr(CS) for that lick type, and then computed the ratio p(x|CS) Pr(CS) / p(x). This ratio is the posterior probability Pr(CS|x). Finally, we computed the error-induced spatial pattern of complex spikes by subtracting the posterior probability Pr(CS|x_s) (i.e., successful licks) from the same ratio for the unsuccessful licks, i.e., Pr(CS|x_u) (Fig. 8C).

The results revealed that when the lick was unsuccessful, the CS events were more likely after the tongue had touched the far end of the tube. To confirm this error-induced effect in another way, we plotted the CS rates as a function of time with respect to when the tongue touched the tube. For the successful licks, tube touch occurred when the tongue crossed the tube’s edge. In this case, the CS rates following tube touch were depressed (Fig. 8D). In contrast, for the unsuccessful licks, licks the tube touch was in error, and the CS rates following this event showed a sharp increase (100 ms period following tube touch, CS rate in unsuccessful vs. successful licks, paired t-test, t(459)=8.09 p=5.3x10E-15).

Thus, when the food was inside the tube and the tongue successfully entered it, tube touch did not elicit complex spikes. However, when the food was inside the tube and the tongue missed the entrance, now the tube touch elicited an increased rate of complex spikes. Thus, the CS rates increased when tube touch signaled a trajectory error.

Error in lick trajectory induced complex spikes. **A**. Successful licks (tongue entered the tube and touched the food). The left figure shows the spatial likelihood at 25 ms before the CS event. The right figure shows the probability of being at a spatial location. **B**. Unsuccessful licks (tongue did not enter the tube and collided with the tube edge). **C**. Spatial pattern of the error induced complex spikes (difference in the posterior probability of complex spikes for the unsuccessful and successful licks). **D**. Complex spike rates following tube touch for successful and unsuccessful licks.

**8) Was the distance of the tongue tip to the mouth measured linearly from the tongue tip at max displacement to the mouth, or the curved distance defining the length of the tongue? Was lick tortuosity affected during any of these CS suppression conditions in Fig 3? More details on the defined aspects of lick kinematics would be helpful.**

The distance was linearly measured. In the added videos we also added kinematic features that show the various measures for each lick as a function of time.

**9) Recent studies in mice have implicated cortical (Bollu, Ito et al., 2021, Nature; Xu et al, 2022, Nature) and collicular (Rossi et al, 2016; Li and Sabatini, 2021; Ito, Gao et al., 2024, bioRxiv) circuits for tongue control, and these studies are not cited in the paper. These studies describe both premotor signals and signals for errors in the motor cortex (missing a water spout) as well as unexpected touch events on the tongue.** **While the authors included a brief paragraph in the Discussion regarding multi-regional interactions among the motor cortex, superior colliculus and cerebellum, a paragraph discussing 1) whether signals similar to those reported in the two papers described above are predicted to be observed in marmosets, and 2) if so, how these signals could be integrated with the cerebellar signals described in the current manuscript, would be helpful in integrating results discovered across species in the manuscript.**

Thank you for the excellent suggestion. We added the following to the Discussion.

*A cortico-cerebellar network for control of the tongue*

As the subject prepared to initiate a licking bout, the P-cells exhibited a ramping activity. Previous reports have noted that during this period there is ramping activity in the tongue regions of the motor cortex (50), as well as in the fastigial (50) and the dentate nuclei (51). Inhibiting the motor cortex in mice prevents both the onset, and the termination of the licking bout (52), suggesting that both are active processes that are cortically mediated. Inhibiting the fastigial during the ramping period disrupts planning of the movement and removes the selectivity that the motor cortical cells exhibit for direction of tongue movements (50), while inhibiting the dentate disrupts the ramping activity in the motor cortex (51). Similarly, exciting the P-cells in the vermis abolishes the ramping activity in the motor cortex during the delay period of a decision-making task (53). This implies that as one prepares to initiate a movement, the rising activity in the motor cortex is controlled via a loop through the cerebellum.

Minor

**Time lag between SS rates between ipsi vs. contra. Is this a feature also observed in SS rates during saccades in PCs?**
We did not find significant differences between SS lag with respect to the lick for ipsilateral vs. contralateral licks (Fig. 6A).

**Did CS activity across PCs (Fig 2c) differ depending on contra vs. ipsi licks?**
Yes. The CS rates were generally larger for ipsilateral licks (Fig. 6A).

**Figure image quality was not preserved upon insertion, making them blurry when zooming in. This is particularly noticeable in Fig 3 when looking at the spike rates and kinematic changes.**
Fixed.

**Fig. 3 took me a while to understand. For the heatmaps in Figs 3a, d and g, it would be helpful to clearly define that the plots show the difference between suppressed and non-suppressed (e.g., Dist. to mouth (Δ supp./not supp.)) within the figure itself.**
Done

**It would be helpful for all panels in Figs. 3b, e, and h, to have the color of the single 'n' in 'n - (n -1)' yellow as in the top left panel of each figure for the reader.**

Done
