# Supplementary material for: Purkinje cells of the cerebellum control deceleration of tongue movements
Source: PLoS Biol. 2025 Apr 10;23(4):e3003110. doi: 10.1371/journal.pbio.3003110 (PMC11984719; doi:10.1371/journal.pbio.3003110)
Supplement: Attachment — Submitted filename: Response to reviewer comments 2.docx [file pbio.3003110.s022.docx]

Response to reviewer comments

Reviewer #2: In this revision, my previous major concerns are all addressed with additional data and analysis. However, I found that some of these additions are not fully integrated. In particular, even though they have demonstrated that non-CS SS pauses are sufficient to produce hypermetria just as CS-driven SS pauses, the abstract (lines 35-36) still describes the phenomenon as if it is specific to climbing fiber inputs (and therefore CSs). A similar omission can be seen at the beginning of the Discussion (lines 398-400).

Done. We updated the abstract and discussion to note that the effects were present for all SS pauses, regardless of their origin.

In addition, I previously provided some references about non-CS SS pauses (Shin and De Schutter, J Neurophys, 2006; Hong et al., eLife 2015; Brown et al., Neuron 2024, etc.), but was surprised to find that none of them is cited in this revision. I am not claiming that the authors are obliged to cite those works, but wouldn't it be nice to acknowledge prior work on SS pauses, rather than completely ignoring them altogether? Also, given their explanation referring to molecular layer interneurons, I think that Lee et al., "Circuit mechanisms underlying motor memory formation in the cerebellum", Neuron, 2015 is very relevant. I hope that the authors take my suggestion seriously.

We added the references associated with previous work on long SS pauses.

Reviewer #3 (Jesse Goldberg): This is a thorough revision that addressed our concerns, and I think that the response to reviewer 2 was substantial. The association of CS with errors is new and interesting in this revision. Overall this is a fantastic paper - a novel behavioral paradigm, precise kinematic tracking, and identification of important cerebellar signals for tongue control.

There is a mis-citation here. Ref 56 and Bollu et al, 2021 (not cited) both show that licks can be initiated during orofacial motor cortex photoinhibition in mice. This should be fixed.

The citations were fixed. Thank you.
